# Efficient purification of wastewater by applying mechanical force and BaCO_3_/TiO_2_ and BaTiO_3_/TiO_2_ piezocatalysts†

**DOI:** 10.1039/d1ra07742b

**Published:** 2021-11-18

**Authors:** Omid Amiri, Haval Aziz Ahmed, Abdulla Ahmed Abdan, Peshawa H. Mahmood, Masoud Salavati-Niasari

**Affiliations:** Department of Chemistry, College of Science, University of Raparin Rania Kurdistan Region Iraq oamiri@uor.edu.krd +9647700581175; Institute of Nano Science and Nano Technology, University of Kashan Kashan P. O. Box. 87317-51167 I. R. Iran salavati@kashanu.ac.ir +98 31 55913201 +98 31 55912383

## Abstract

In typical advanced oxidation catalysis, a semiconductor should have a robust capacity to generate separated electron–hole pairs on a material's surface under irradiation of photons with energy more than the material's bandgap. However, rapid charge carrier recombination and low photon to current yield of semiconductor photocatalysts and low percentages of UV light in sunlight leads to a low level of photocatalytic efficiency for practical application. Mechanical energy is a natural energy that can be considered as a form of rich, clean and renewable energy which can be harvested by using piezoelectric materials. Here, we developed BaCO_3_/TiO_2_ and BaTiO_3_/TiO_2_ composites as mechanical harvesting materials to decontaminate pollutants. Results showed that BaCO_3_ has a great effect on the piezocatalytic activity of products. The control sample (sample without Ba) only degraded 11.2% of Acid Red 151 (AR151) , while the sample containing Ba degraded 96.7% of AR151. Besides, the effects of several parameters, including the natural surfactant, reaction time and temperature, calcination, and ultrasonic power and pulse on the catalytic activity of the as-prepared piezocatalysts were studied. Results showed that it is possible to degrade 99.1% of AR151 by controlling ultrasonic parameters during 2 h of mechanical energy force.

## Abstract 2

In typical advanced oxidation catalysis, a semiconductor should have a robust capacity to generate separated electron–hole pairs on a material's surface under irradiation of photons with energy more than the material's bandgap.

## Graphical abstract

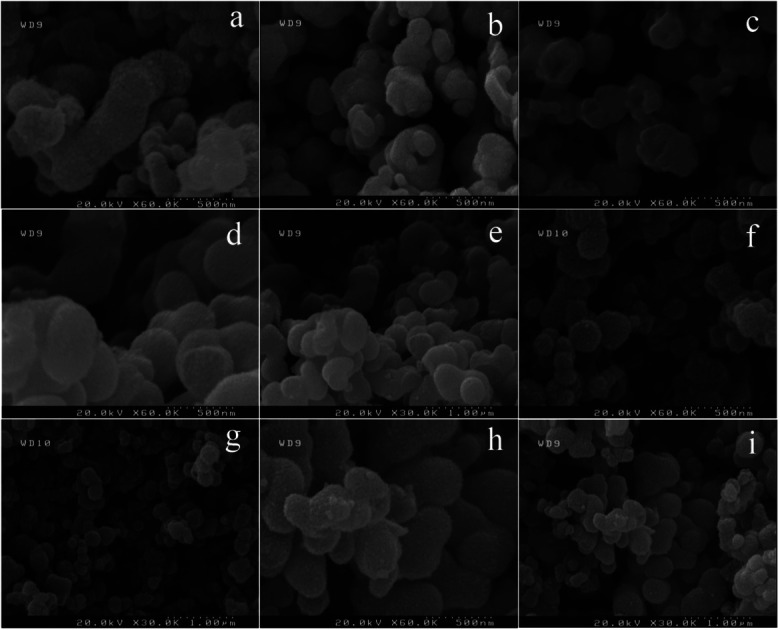

## 1. Introduction

The need for environmental protection continues to rise as a result of universal population growth and fast manufacturing development. High stability organic contaminants in water make it difficult to treat. Such organic contaminants can lead to serious health problems and ecological issues. In recent decades, research on applying advanced oxidation technologies (AOT) to treat wastewater has continued to rise.^1–3^ In AOT, semiconductor materials have been used for decomposition of organic contaminants, whereby strong oxidizing radicals are generated on illumination in visible to ultraviolet light wavelength region. Finally, these free radicals react with dye molecules.^4,5^ In typical advanced oxidation catalysis, semiconductor should have a robust capacity to generate separated electron–hole pairs on material surface under radiation of photons with energy more than material bandgap. However, rapid charge carrier's recombination and low photon to the current yield of semiconductor photocatalysts, low percentages of UV light in sunlight leads to a low level of photocatalytic efficiency for practical application. Therefore, researchers look for alternative clean and renewable energy to treat wastewater.

Mechanical energy is a natural energy that can be considered as a form of rich, clean and renewable energy which can be harvested by using piezoelectric materials. In piezoelectric materials, such as perovskite BaTiO_3_, an applied external force or stress causes slight displacement of the charge centers of cations and anions. This displacement creates allied dipole moments which results in an inner electric field (piezoelectric potential).^6–8^ This inner electric field can drive free charge carriers to flow through external circuits in linear electromechanical behavior, which extensively used for the purpose such as active sensing, mechanical energy harvesting, electrical actuating, and self-powered system.^9–11^

Recently, piezoelectricity has been applied as an effective method to enhance the degradation efficiency of photocatalysis.^12–14^ For instance Xinyu Xue *et al.* reported improvement of degradation efficiency of methylene blue by coupling piezoelectric and photocatalytic properties of ZnO nanowires.^15^ In other research published in 2019, Shuya Xu *et al.* reported enhanced Photocatalysis of BaTiO_3_ by piezotronic effect.^6^ The enhancement comes from the fact that the electric field can promote the separation of photo-generated charge carriers.

In the last few years, piezoelectric materials were considered as a new class of catalyst for the degradation of organic pollution.^16–23^ For example, Hong *et al.* illustrated that the degradation of acid orange 7 in the presence of piezoelectric BaTiO_3_ micro-dendrites and by applying ultrasonic wavelength.^16^ Same results reported by He Lin *et al.* where treated water containing acid orange 7 using piezoelectric effect in Pb(Zr_0.52_Ti_0.48_)O_3_ (PZT) fibers.^18^ Lv *et al.* studied the effect of metal ions and BaTiO_3_ in the presence of metal ion on the generation of *in situ* H_2_O_2_ which led to facile production of hydroxyl radical with strong oxidative power, leading to decomposition of organic pollutants.^19^ Jyh Ming Wu *et al.* illustrated that MoS_2_ and MoSe_2_ nanoflowers show an ultrahigh piezocatalytic activity in dark under continuous ultrasonic vibration. Very recently, Jiang Wu studied the effect of annealing temperature on the piezoelectric activity of BaTiO_3_.^24^ Piezocatalysis is especially attractive compared to the current catalytic technologies such as photocatalysis and electrocatalysis due to the ability to use the prevalent mechanical vibration and to reduce the dependence on other conditions, such as light and electricity.^25–27^ Up to now, piezomaterials were rarely used to treat water.

Here, we prepared BaCO_3_/TiO_2_ and BaTiO_3_/TiO_2_ composites by hydrothermal method. The effect of vibration pulse and capping agent on the piezoelectric activity of BaTiO_3_/TiO_2_ composite did not study so far. The effect of vibration power, pulse, time and natural capping agent, including plum, apricot, peach, and melon was studied on the degradation efficiency of wastewater containing Acid Red 151 (AR151).

## 2. Experimental

### 2.1. Material

BaCl_2_·2H_2_O, tetraethyl orthotitanate (TEOT), NaOH and ethanol were purchased from Merck. Natural surfactant including plum, apricot, peach, and melon obtained from fresh fruits.

### 2.2. Synthesis of nanopiezoelectric materials

In a typical synthesis process, 1.07 g of BaCl_2_·2H_2_O was dissolved in deionized water. 3 mL of natural surfactant was added to aqueous solution of barium and stirred for 10 min. In another beaker, 1 mL of tetraethyl orthotitanate was mixed with 10 mL of ethanol and stirred for 5 min. Afterward, ethanol based solution of tetraethyl orthotitanate was added to aqueous mixture of barium and natural surfactant under stirring. Finally, 3 mL of 3 M NaOH solution was added and keep stirring for 10 min. The above mixture transferred to a 200 mL autoclave and heated at 160 °C for 6 h. The obtained precipitate washed twice with water and ethanol and dried for more application. In the synthesis section, we studied the effect of time and heating temperature and natural surfactant on the morphology and catalytic activity of BaTiO_3_/TiO_2_ composite. Experimental detail for the synthesis of piezocatalysts could be found in Table S1.†

### 2.3. Study of piezocatalytic activity

To study the piezocatalytic activity of BaTiO_3_/TiO_2_ composite and the effect of ultrasonic pulse, power and time on its catalytic activity, systematic series of experiments were done. For this, 1 g L^−1^ of BaTiO_3_/TiO_2_ composite was dispersed in 50 mL of 5 ppm Acid Red 151 aqueous solution. Then, the mixture was vibrated by using ultrasonic vibration with different powers, pulses and times. After a certain time, UV-Vis spectrum of sample was measured to pursue the degradation of organic pollution.

### 2.4. Characterization

XRD was measured by using Philips-X'PertPro, an X-ray diffractometer with Ni-filtered Cu K_α_ radiation at the scan range of 10 < 2*θ* < 80. FT-IR analysis was carried out using Shimadzu Spectrophotometer. The morphology and size of samples were observed by field emission scanning electron microscopy (FE-SEM, Mira3 Tescan). 6705 UV-Vis spectrometer, JenWay was used for UV-Vis spectra measurements.

## 3. Results and discussion

**Fig. 1:**
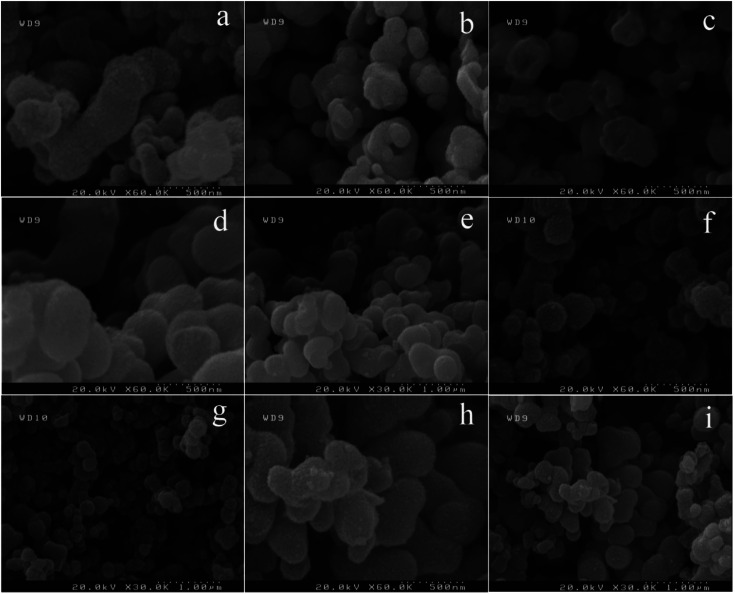
FE-SEM images of as-prepared catalysts without surfactant (a) and by using plum (b) and (c), apricot (d) and (e), peach (f) and (g), and melon (h) and (i) as a capping agent.

Effect of natural surfactant on the morphology of BaCO_3_/TiO_2_ and BaTiO_3_/TiO_2_ composites were studied by using plum, apricot, peach, and melon juice and were compared with the sample without surfactant (Fig. 1). In this regard, BaTiO_3_/TiO_2_ composite was first prepared at 160 °C for 6 h without adding surfactant to the precursor (sample 1). In this synthesis condition, very small nanoparticles were assembled and formed peanut-like microstructures with 200–500 nm in size (Fig. 1a). In the preparation stage for the next sample, 3 mL of plum juice was used as a capping agent (sample 2) and smaller and more spherical shape microstructures were achieved (Fig. 1b and c). As shown in Fig. 1d and e, using 3 mL of apricot juice as a capping agent led to produce a more uniform microstructures in size and shape (sample 3). Nanoparticles of BaTiO_3_/TiO_2_ were assembled and formed spherical shape structures with 300 nm in diameter. When peach was used in the synthesis of BaTiO_3_/TiO_2_ nanostructures (sample 4), the size of assembled microspheres decreased to 150–250 nm. They had a completely spherical shape (Fig. 1f and g). As the last capping agent, melon was used to prepare BaTiO_3_/TiO_2_ catalysts (sample 5) which related FE-SEM images are presented in Fig. 1h and i. Irregular assembled microstructures were formed.

In the next step, we studied the effect of hydrothermal temperature (140, 160, and 180 °C) on the morphology of final products. Interestingly, nanoparticles did not have enough energy to assemble and form microsphere structures when the reaction temperature was 140 °C (Fig. S1a and b†). In this case, very small and uniform nanoparticles with size below 20 nm were formed (sample 6). As discussed above, when the reaction temperature was 160 °C (sample 1), nanoparticles assembled and formed peanut-like (Fig. 1a), microstructure with 200–500 nm in size (Fig. 1a). More increasing the reaction temperature to 180 °C (sample 7) in the synthesis of BaTiO_3_/TiO_2_ composite led to form the mixture of nanoparticles and aggregated nanoparticles (Fig. S1c and d†).

Reaction time was studied as the last parameters that could affect the morphology and piezoelectricity (Fig. S2a–d†). BaTiO_3_/TiO_2_ composites were prepared in three reaction time including 4, 6, and 8 h. It seems 4 h is not enough time to form peanut microstructures (Fig. S2a and b†). Fig. S2a and b† showed that nanoparticles start to assemble and form peanut structures. It could still see peanut structures somewhere (indicated by red circle). As shown in Fig. 1a, peanut-like microstructures with 200–500 nm in size were when the reaction time was 6 h. Keeping the autoclave in the oven for 8 h did not show a significant effect. However, more spherical microstructures of BaTiO_3_/TiO_2_ were formed when the reaction time was 8 h (Fig. S2c and d†).

**Fig. 2:**
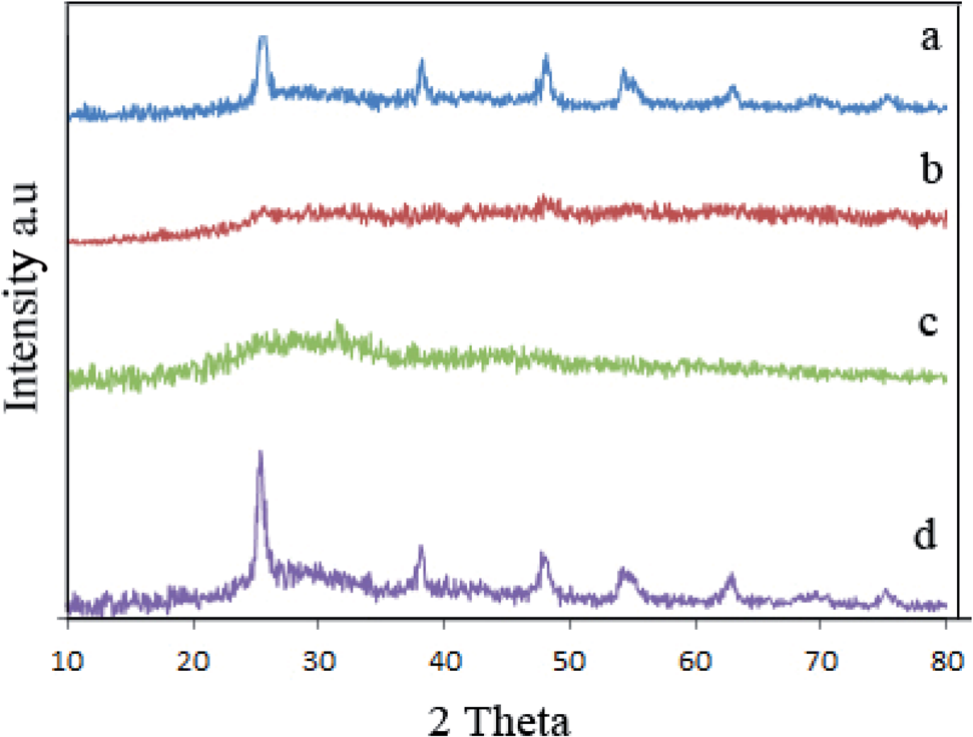
XRD pattern of sample 1 (a), sample 2 (b), sample 3 (c), and sample 6 (d) before calcination.

Fig. 2 illustrated XRD pattern of samples 1, 2, 3, and 6 that indicated the effect of surfactant and temperature on the crystalline structure of piezocatalysts. XRD pattern for piezocatalysts prepared without surfactant is presented in Fig. 2a which exhibited TiO_2_ and amorphous BaCO_3_ were formed. By adding plum and apricot as a capping agent, amorphous structures were formed (Fig. 2b and c). Fig. 2d showed the effect of reaction temperature on the composition of final products (sample 6). By comparing XRD pattern of sample 1 (prepared at 160 °C) and sample 6 (prepared at 140 °C), we figured out that increasing reaction temperature from 140 °C to 160 °C improves the crystallinity of products. Samples 1, 2 and 3 were calcinated to prepare BaTiO_3_/TiO_2_ composites. Fig. S3a–c† represented XRD pattern of samples 1–3 after calcination at 750 °C for 2 h. As shown in Fig. S3,† all samples were crystalized and BaTiO_3_/TiO_2_ was formed.

**Fig. 3:**
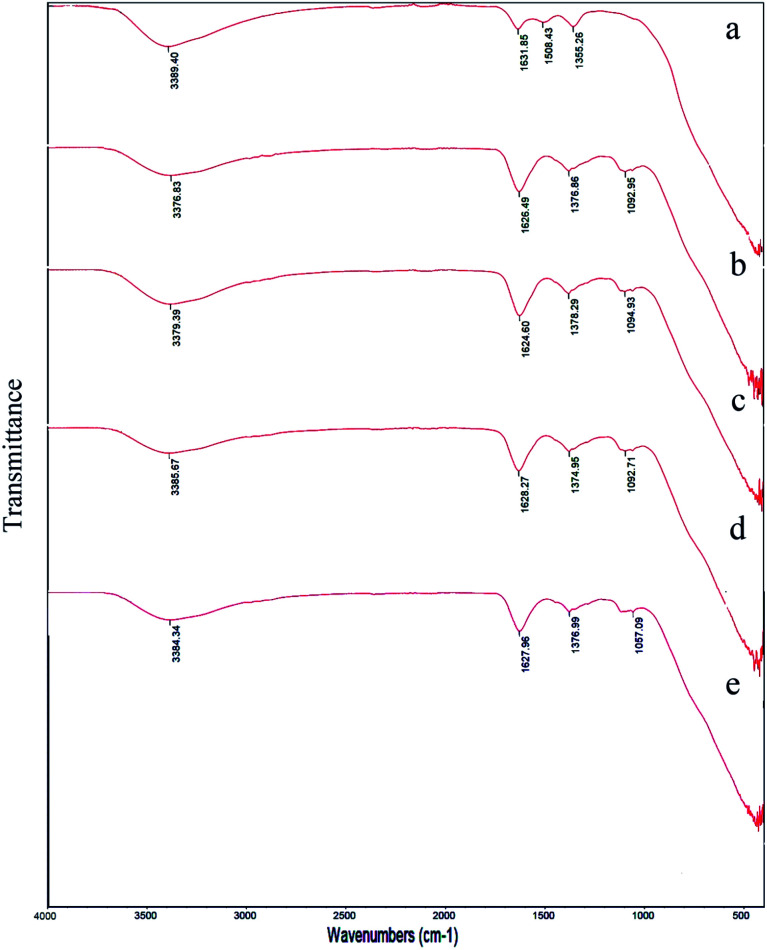
FT-IR spectra of sample 1 (a), sample 2 (b), sample 3 (c), sample 4 (d), and sample 5 (e) before calcination.

**Fig. 4:**
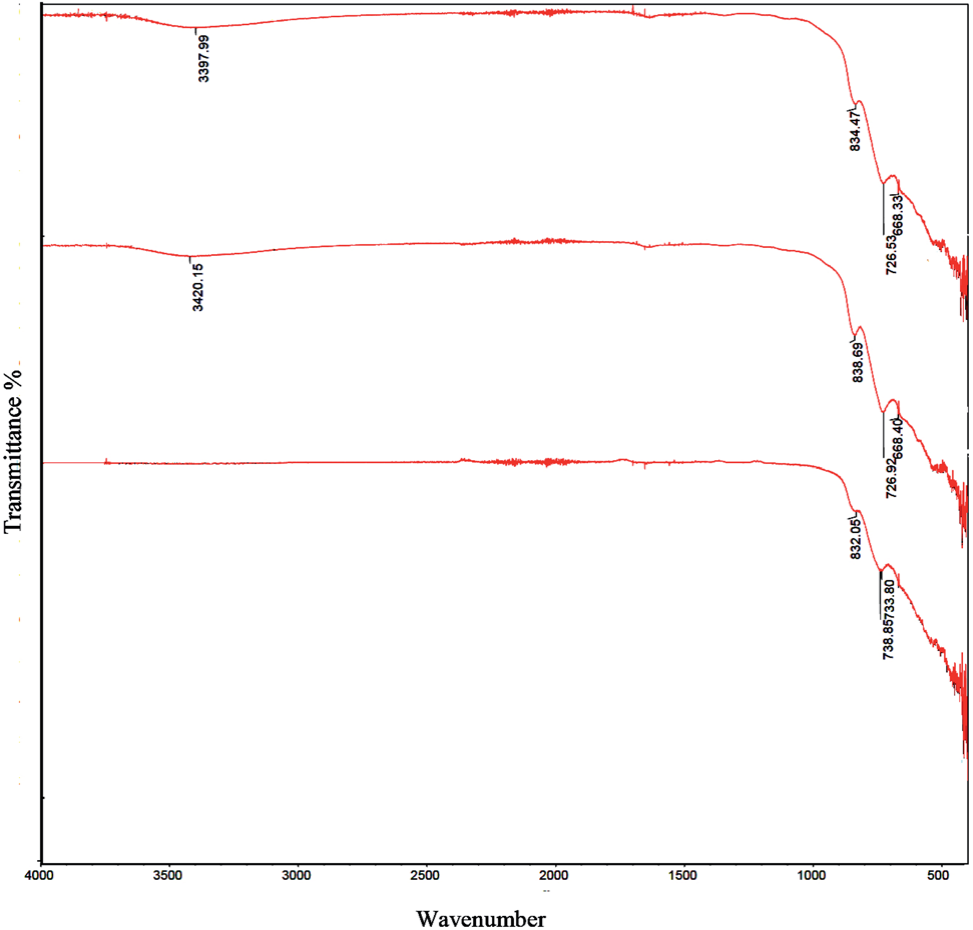
FT-IR spectra of sample 1 (a), sample 2 (b), and sample 3 (c), sample 4 (d), and sample 5 (e) after calcination at 750 °C for 2 h.

Fig. 3 and 4 showed FT-IR spectra of samples 1–5 before calcination and samples 1–3 after calcination, respectively. FT-IR of samples 1–5 approved the presence of BaCO_3_ before calcination (Fig. 3a–e). As presented in Fig. 3a–e, all samples presented several peaks and bands associated to carbonate vibrations.^28,29^ The broad peak at ∼1374–1500 cm^−1^ could be assigned to asymmetric stretch of carbonate CO_3_^−^, the peak at 1624–1631 cm^−1^ and peak at 1060 cm^−1^ were related to an organic carbonate and symmetric C–O stretching vibration.^28–34^ Results verified that there were significant amounts of barium carbonate before calcination. Fig. 4a–c showed that peaks related to BaCO_3_ disappeared after calcination. It seemed during calcination BaCO_3_ reacts with TiO_2_ and produces BaTiO_3_ and excess TiO_2_ to create our second compound in BaTiO_3_/TiO_2_ composite.

### 3.1. Catalytic activity test: effect of BaCO_3_ on decontamination efficiency

**Fig. 5:**
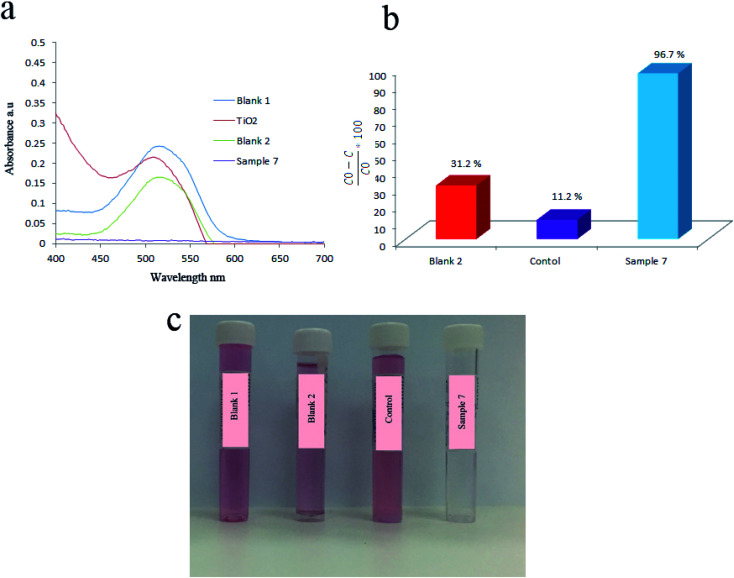
(a) UV-Vis spectra of initial AR151 (blank 1), AR151 after treatment with TiO_2_ (red curve), bare ultrasonic (blank 2), and sample 7. (b) Degradation efficiency by using bare ultrasonic, TiO_2_ (control sample) and sample 7. (c) Photo of blank 1, blank 2, control and sample 7 after 2 h vibration and centrifuging.

BaCO_3_ had a great effect on piezocatalytic activity of products. This was approved by comparing the piezocatalytic activity of sample without Ba that was labeled as control and sample 7 which containing Ba. For this, 1.07 g of BaCl_2_·2H_2_O was first dissolved in deionized water. In another baker, 1 mL of tetraethyl orthotitanate was mixed with 10 mL of ethanol and stirred for 5 min. Afterward, ethanol based solution of tetraethyl orthotitanate was added to aqueous mixture of barium under stirring. Finally, 3 mL of 3 M NaOH solution was added and kept stirring for 10 min. The above mixture transferred to 200 mL autoclave and heated at 180 °C for 6 h. To prepare the control sample, the above steps were repeated by elimination of BaCl_2_·2H_2_O. Acid Red 151 (AR151) was used as an organic pollution to study the piezoelectric activity of all samples. Here, the degradation of AR151 was also studied by ultrasonic vibration and without catalysts that labeled as blank 2. The initial dye solution was labeled as blank 1. The results are presented in Fig. 5 and Table 1. Fig. 5a and b showed UV-Vis spectrum of AR151 and degradation efficiency, while Fig. 5c showed the photo of blank 1, blank 2, control and sample 7 after 2 h vibration and centrifuging. Results were interesting. The degradation reaction dramatically decreased in the sample without Ba. The control sample only degraded 11.2% of AR151, while the sample containing Ba degraded 96.7% of AR151. Such huge enhancement approved the effect of barium carbonate on the piezoelectricity of an as-prepared catalyst. Fig. 5 also showed that 31.2% of AR151 was degraded when ultrasonic vibration with 100 W in power was applied for 2 h without the piezoelectric catalysts.

**Table 1:** Degradation efficiency for AR151 when bare ultrasonic, TiO_2_ and TiO_2_/BaCO_3_ was used as piezocatalyst

Catalyst	Vibration time (h)	Vibration power (W)	Pollutant	Degradation efficiency (%)
Control (TiO_2_)	2	100	AR151	11.2
Blank 2 (bare ultrasonic)	2	100	AR151	31.2
TiO_2_/BaCO_3_	2	100	AR151	96.7

### 3.2. Effect of the type of surfactants on catalytic activity

**Fig. 6:**
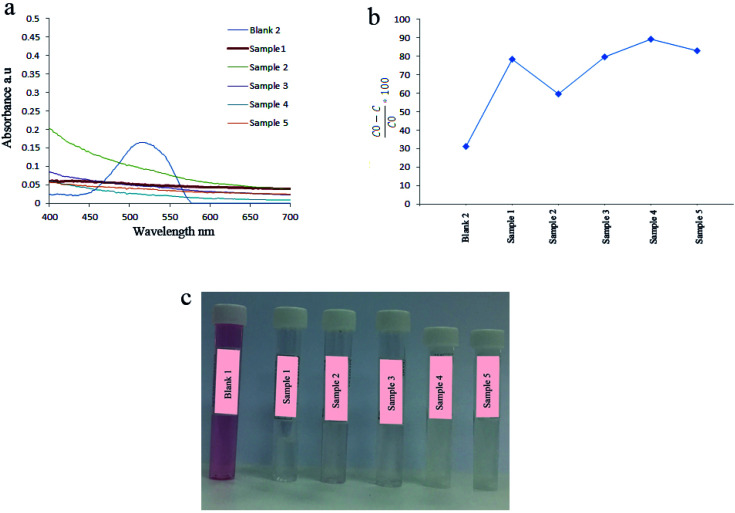
(a) UV-Vis spectra of AR151 after ultrasonic vibration without catalysts (blue curve), and with sample 1 (red curve), 2 (green curve), 3 (purple curve), 4 (light blue), and 5 (orange curve). Related degradation efficiencies (b) and their solution's photos (c).

In this section, the effect of several parameters including a natural surfactant, reaction time and temperature, calcination, ultrasonic power, pulse on the catalytic activity of as-prepared piezocatalysts were studied. The effect of bare ultrasonic waves and using natural surfactants during the synthesis of catalysts on the degradation of AR151 are shown in Fig. 6 and Table 2. Degradation results showed that 31.2% of AR151 was degraded when ultrasonic vibration with 100 W in power was applied for 2 h without the piezoelectric catalysts. In this situation, degradation energy came from acoustic cavitation which was the formation, growth, and implosive collapse of bubbles in a solvent.^3,35,36^ When bubbles were collapsed huge energy was released and this released energy could produce some hydroxyl radicals.^35,36^ These radicals were responsible for the degradation of 31.2% of AR151 in absence of piezocatalysts. When nanopiezocatalysts were used, 78.4% of AR151 was degraded in the same ultrasonic time and power (sample 1). This huge enhancement (47.2%) is because of strong piezoelectricity of as-prepared nanocatalysts. Using natural surfactants in the synthesis of piezocatalysts had a significant effect on the degradation of AR151. As can be seen in Fig. 6, the degradation efficiency of 59.7% was achieved when plum was used as a capping agent in the synthesis of BaTiO_3_/TiO_2_. In the case of using apricot, 79.6% of AR151 was degraded which was close to degradation efficiency of bare BaTiO_3_/TiO_2_. When peach and melon were used as a capping agent in the synthesis of BaTiO_3_/TiO_2_ composites, 89.2% and 83.0% of AR151 were decomposed during 2 h ultrasonic vibration, respectively. Results showed that applied plum as a capping agent decreases piezoelectricity and degradation efficiency while using peach and melon increase piezoelectricity and degradation efficiency. According to XRD results, using plum and apricot led to decrease crystallinity. When plum and peach were used, amorphous BaTiO_3_/TiO_2_ was achieved. Therefore, low degradation efficiencies (59.7 and 79.6%) were achieved.

**Table 2:** Decontamination results by applying sample 1–5 as catalysts

Catalyst	Vibration time (h)	Vibration power (W)	Pollutant	Degradation efficiency (%)
Sample 1	2	100	AR151	78.4
Sample 2	2	100	AR151	59.7
Sample 3	2	100	AR151	79.6
Sample 4	2	100	AR151	89.2
Sample 5	2	100	AR151	83.0

### 3.3. Effect of calcination on decontamination efficiency

**Fig. 7:**
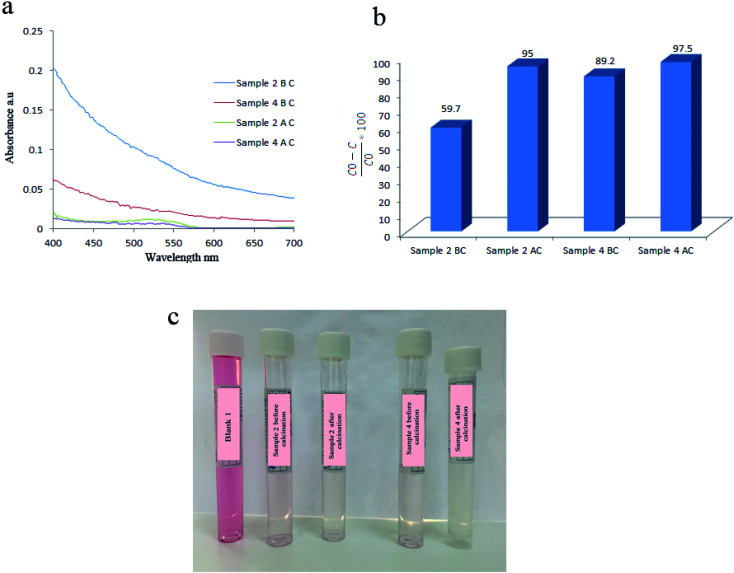
Comparing degradation efficiency of sample 2 and 4 before and after calcination at 750 °C for 2 h: their UV-Vis spectra (a), degradation efficiencies (b), and solution's photos (c).

To study the effect of calcination on degradation efficiency, samples 2 and 4 were calcinated at 750 °C for 2 h and were used as piezocatalysts to degrade AR151. It was observed that degradation efficiency increased to 95.0% and 97.5%, respectively (Fig. 7 and Table 3). Calcination affects degradation efficiency because it changed the composition of final products. As mentioned in Fig. S3,† BaTiO_3_/TiO_2_ was obtained by calcination of samples at 750 °C for 2 h.

**Table 3:** Effect of calcination of catalyst on the decontamination efficiency of AR151

Catalyst	Vibration time (h)	Vibration power (W)	Pollutant	Degradation efficiency (%)
Sample 2 BC	2	100	AR151	59.7
Sample 2 AC	2	100	AR151	95.0
Sample 4 BC	2	100	AR151	89.2
Sample 4 AC	2	100	AR151	97.5

### 3.4. Effect of reaction temperature and time on decontamination efficiency

**Fig. 8:**
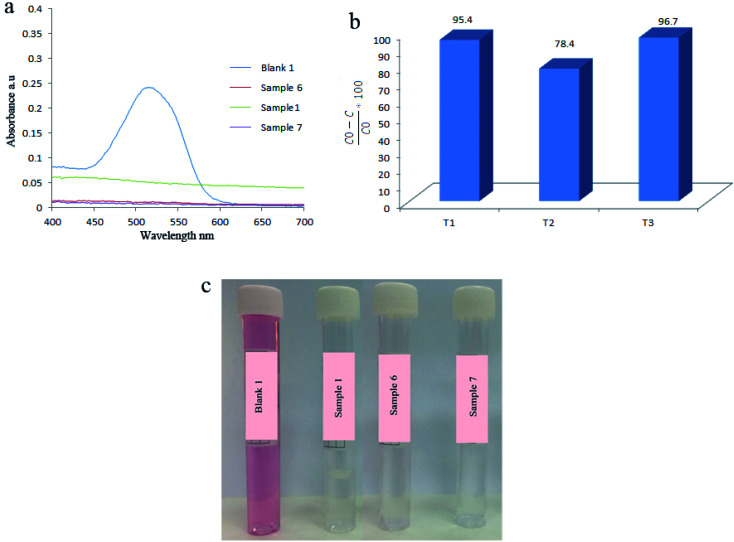
(a) UV-Vis spectra of initial AR151 (blank 1) by using sample 1, 6, 7. Related degradation efficiencies (b) and their solution's photos (c). *T* refers to the reaction temperature: *T*_1_ = 140 °C, *T*_2_ = 160 °C and *T*_3_ = 180 °C.

Afterward, the effect of hydrothermal temperature during the synthesis of piezoelectric catalysts was investigated. Related results are presented in Fig. 8 and Table 4 which showed when hydrothermal temperature was 140 °C, 95.4% of AR151 was degraded during 2 h of ultrasonic vibration. By changing hydrothermal temperature to 160 °C, degradation efficiency changed to 78.4%. By increasing hydrothermal temperature to 180 °C, degradation efficiency went up to 96.7%. In the first step, degradation efficiency decreased by 17.4% when hydrothermal reaction increased 20 °C (from 140 to 160 °C). Degradation efficiency increased by 22.7% (from 78.8 to 96.7%) when hydrothermal temperature increased from 160 to 180 °C (20 °C). These were unexpected results. It was expected that sample prepared at 140 °C showed lower degradation efficiency compared to that prepared at 160 °C because increasing reaction temperature increased the crystallinity. XRD result approved this and the sample prepared at 160 °C showed higher crystallinity (its peaks had higher intensity). Morphology could explain these unexpected results, as well. As can be seen in Fig. 1 and S1,† these two samples (1 and 6) had different morphologies. It seemed uniform nanoparticles show higher piezocatalytic activity compared to irregular peanut-like structures.

**Table 4:** Effect of reaction temperature during synthesis of catalyst on the degradation efficiency of AR151

Catalyst	Vibration time (h)	Vibration power (W)	Pollutant	Degradation efficiency (%)
*T*_1_	2	100	AR151	95.4
*T*_2_	2	100	AR151	78.4
*T*_3_	2	100	AR151	96.7

**Fig. 9:**
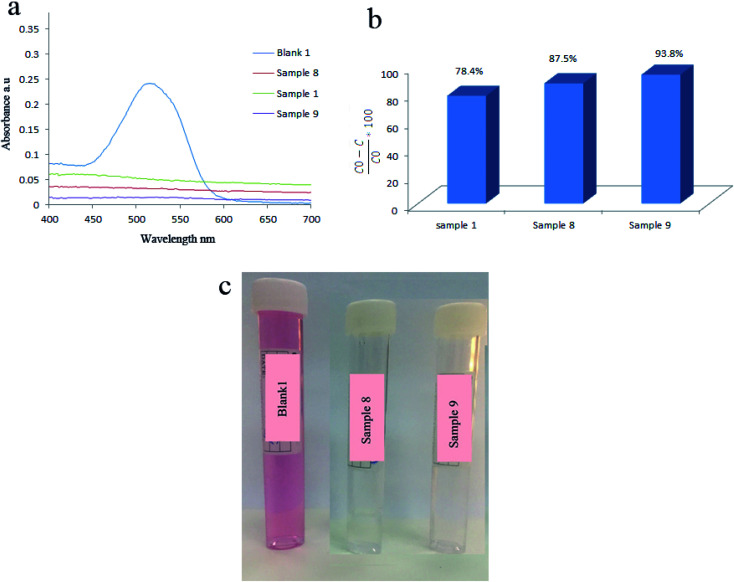
Effect of hydrothermal reaction time on decontamination of AR151: UV-Vis spectra (a), degradation efficiencies (b), and solution's photos (c) of AR151 when sample 1, 8 and 9 were used as piezocatalyst.

Hydrothermal reaction time was another parameter that determines the piezoelectricity and degradation efficiency. Fig. 9 showed that applying BaTiO_3_/TiO_2_ prepared at 160 °C for 4 h leads to degrading 87.5% of AR151. By changing the reaction time to 6 h in the synthesis of piezocatalysts, degradation efficiency decreased to 78.8%. Degradation efficiency increased to 93.8% by more increasing the reaction time in the synthesis of catalysts (8 h). In this case, morphology could explain why sample 8 showed the higher degradation efficiency compared to sample 1, as well. Sample 8 contained aggregated nanoparticles. Based on the above results, samples with more symmetrically morphology showed higher piezocatalytic activity.

### 3.5. Effect of vibration power and pulse on decontamination yield

**Fig. 10:**
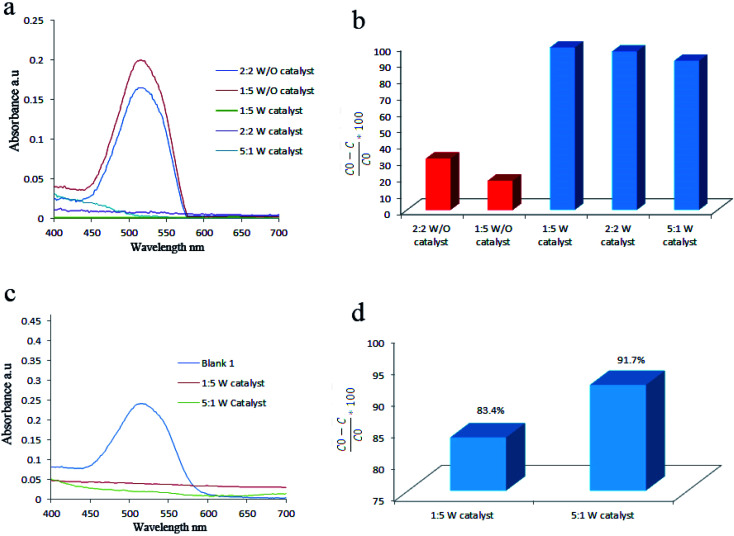
Effect of ultrasonic pulse and power on decontamination of AR151: UV-Vis spectra, power 100 W (a), degradation efficiencies, 100 W (b), UV-Vis spectra, power 300 W (c) and degradation efficiencies, 300 W (d).

In another section, the effect of ultrasonic power and pulse was studied on the degradation efficiency of AR151 (Fig. 10a, b and Table 5). Results were interesting. Three different pulses including 2 : 2 on : off, 1 : 5 on : off, and 5 : 1 on : off were studied in two different ultrasonic powers (100 and 300 W). Degradation efficiency of AR151 without catalysts under ultrasonic wave with 100 W in power decreased from 31.2% to 17.8% when ultrasonic pulse changed from 2 : 2 on : off to 1 : 5 on : off. This could happen because the time of vibration was not enough for transient bubbles to go through liquid.^37^ Besides, only 10 min vibration was applied when the pulse was 1 : 5 (on : off) during 1 h sonication which was much lower than 30 min vibration when the pulse was 2 : 2 (on : off). It means there were more H and OH radicals when the pulse is 2 : 2 (on : off). Therefore, degradation efficiency increased when the pulse was 2 : 2 for the sample without catalysts. In the case of using piezocatalyst, reverse behavior was observed. By adding 1 g L^−1^ of piezocatalyst and repeating the test in the same condition (pulse: 1 : 5 on : off, power: 100 W), 99.1% of AR151 was degraded which was interesting. Meanwhile, the degradation efficiency of 96.7% was achieved when piezocatalysts were used and pulse was 2 : 2 (on : off). It seems piezocatalysts needed time to back to the ground state to show piezoelectricity again and produce more active radicals. Here, it was defined a state that positive and negative charge symmetrically were dispersed as the ground state. In this regard, piezocatalysts had enough time to back to the ground state when the pulse is 1 : 5 (on : off) therefore, it could prepare more radicals in the next vibration pulse and show higher degradation efficiency. When pulse changed to 5 : 1 (on : off), degradation efficiency of AR151 decreased to 91.0% which could happen because of the same reason. When vibration continually applied for 5 s and paused for 1 s, piezocatalysts had no time to back to the ground state therefore, showed lower efficiency compared to those with 2 : 2 and 1 : 5 on : off.

**Table 5:** Effect of vibration pulse and power on the decontamination yield

Catalyst	Vibration time (h)	Vibration power (W)	Pulse (S)	Pollutant	Degradation efficiency (%)
W/O catalyst	2	100	2 : 2	AR151	31.2
W/O catalyst	2	100	1 : 5	AR151	17.8
W catalyst	2	100	1 : 5	AR151	99.1
W catalyst	2	100	2 : 2	AR151	96.7
W catalyst	2	100	5 : 1	AR151	91.0
W catalyst	2	300	1 : 5	AR151	83.4
W catalyst	2	300	5 : 1	AR151	91.7

In another try, the degradation efficiency of piezocatalysts was studied in different ultrasonic powers (300 W). The results of this batch are presented in Fig. 10c and d. According to the results, applying ultrasonic wave with pulse 1 : 5 on : off led to degrading 83.4% of AR151. By changing pulse to 5 : 1 on : off, degradation increased to 91.7%. No explanation could be found for this.

### 3.6. Piezocatalytic mechanism

**Fig. 11:**
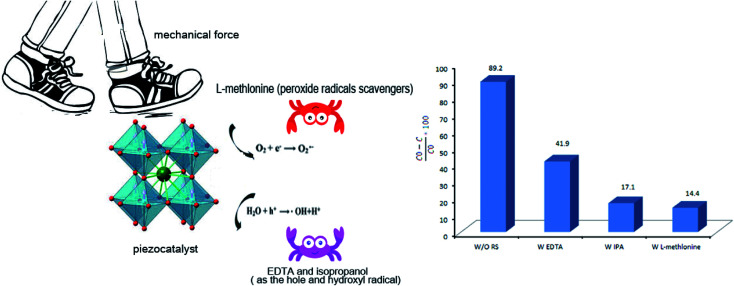
Possible mechanism for decontamination of AR151 by using a piezocatalyst. EDTA, IPA, and l-methlonine as hole (h^+^), hydroxyl radical (˙OH), and peroxide radical (O2˙^−2^) scavengers, respectively.

The radical trapping experiment was carried out by using EDTA, isopropanol, and l-methlonine as hole (h^+^), hydroxyl radical (˙OH), and peroxide radicals (O_2_˙^2−^) scavengers, respectively. As shown in Fig. 11, EDTA, isopropanol, and l-methlonine significantly suppressed the piezoelectric decontamination process. Compared with the absence of EDTA, l-methlonine, and IPA, the decontamination efficiencies decreased from 89.2% to 41.9%, 14.4%, and 17.1%, respectively. So, the result of radical trapping evaluation indicated that the piezo-generated superoxide radicals (˙O_2_^−^), hydroxyl radical (˙OH), and holes (h^+^) played the dominant role in the piezoelectric decontamination of AR151.^38–41^

## 4. Conclusion

In the current research paper, BaCO_3_/TiO_2_ and BaTiO_3_/TiO_2_ were prepared by hydrothermal method. Results showed that BaCO_3_ had a significant effect on the piezodecontamination of TiO_2_. When the control sample prepared without BaCO_3_, the piezocatalytic activity of TiO_2_ dramatically decreased. Piezodecontamination efficiency decreased from 96.7 to 11.2% by elimination of BaCO_3_. BaTiO_3_/TiO_2_ composites could be prepared by calcination of first products at 750 °C for 2 h. BaTiO_3_/TiO_2_ composites showed higher piezodecontamination efficiency compared to BaTiO_3_/TiO_2_ composites. Degradation efficiency increased from 59.7 and 78.4 to 97.5% and 95% by calcination of samples 1 and 2 at 750 °C, respectively. Different parameters such as using natural surfactants, reaction time and temperature, ultrasonic power, and pulse determined the activity of as-prepared piezocatalysts. Results showed that applying plum as a capping agent in the synthesis of catalysts decrease piezoelectricity and degradation efficiency while using peach and melon increase piezoelectricity and degradation efficiency. Since reaction time and temperature controlled the morphology of final products, they showed a significant effect on degradation efficiency. Based on the above results, samples with more symmetrically morphology showed higher piezocatalytic activity. The effect of vibration pulse on piezocatalytic activity was studied. 99.1% of AR151 was degraded when pulse and power were 1 : 5 on : off and 100 W, respectively. Meanwhile, the degradation efficiency of 96.7% was achieved when piezocatalysts were used and pulse was 2 : 2 (on : off). It seemed piezocatalysts need time to back to the ground state to show piezoelectricity again and produce more active radicals. A state was defined that positive and negative charges symmetrically were dispersed as the ground state. In this regard, piezocatalysts had enough time to back to the ground state when the pulse is 1 : 5 (on : off). Therefore, it could prepare more radicals in the next vibration pulse and show higher degradation efficiency. When pulse changed to 5 : 1 (on : off), the degradation efficiency of AR151 decreased to 91.0% which could happen because of the same reason. Finally a possible mechanism was suggested based on the radical trapping experiment.

## Conflicts of interest

There is no conflicts of declare.

## Supplementary Material

RA-011-D1RA07742B-s001
