# Supplementary material for: Efficient purification of wastewater by applying mechanical force and BaCO3/TiO2 and BaTiO3/TiO2 piezocatalysts
Source: RSC Adv. 2021 Nov 18;11(59):37138–49. doi: 10.1039/d1ra07742b (PMC9043623; doi:10.1039/d1ra07742b)
Supplement: RA-011-D1RA07742B-s001 [file RA-011-D1RA07742B-s001.pdf]

Electronic Supplementary Material (ESI) for RSC Advances.
This journal is © The Royal Society of Chemistry 2021

Efficient purification of wastewater by applying mechanical force and $BaCO_3/TiO_2$ and $BaTiO_3/TiO_2$ Piezocatalysts

Omid Amiri [a] *, Haval Aziz Ahmed [a], Abdulla Ahmed Abdan [a], Peshawa H. Mahmood [a], Masoud Salavati-Niasari[b]**

[a] Department of Chemistry, College of Science, University of Raparin, Rania, Kurdistan Region, Iraq
[b]Institute of Nano Science and Nano Technology, University of Kashan, Kashan, P. O. Box. 87317-51167, I. R. Iran
** Corresponding author. Tel: +9647700581175; E-mail address: oamiri@uor.edu.krd (O. Amiri)*
*** Corresponding authors:E-mail address: salavati@kashanu.ac.ir (Prof. M. Salavati-Niasari)*
Tel.: +98 31 55912383; Fax: +98 31 55913201

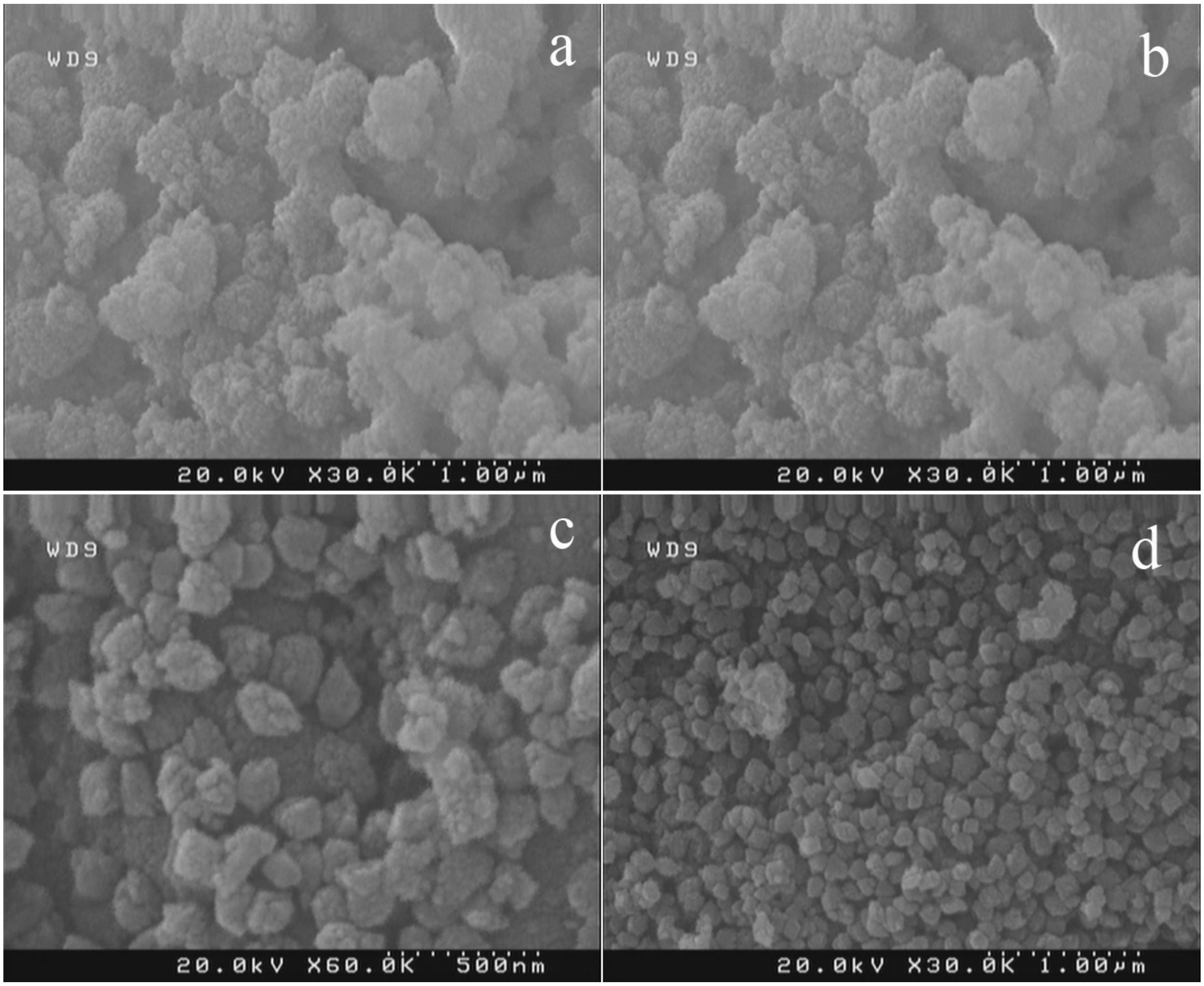

Fig. S1. SEM images for sample prepared at 140 °C (a, b) and 180 °C (c, d).

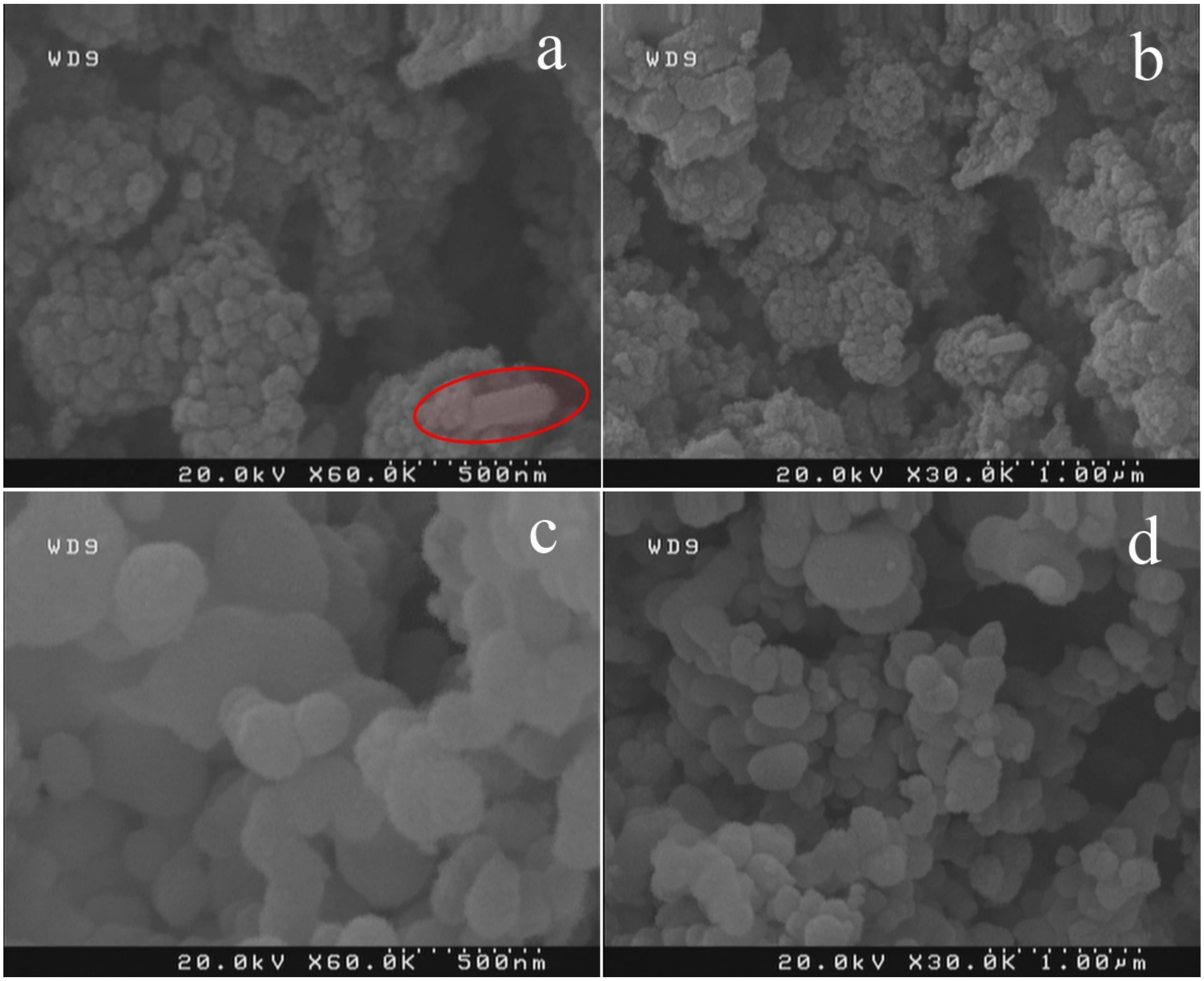

Fig. S2. Effect of reaction time on the morphology of final products: a, b) 4 h and c, d) 8 h.

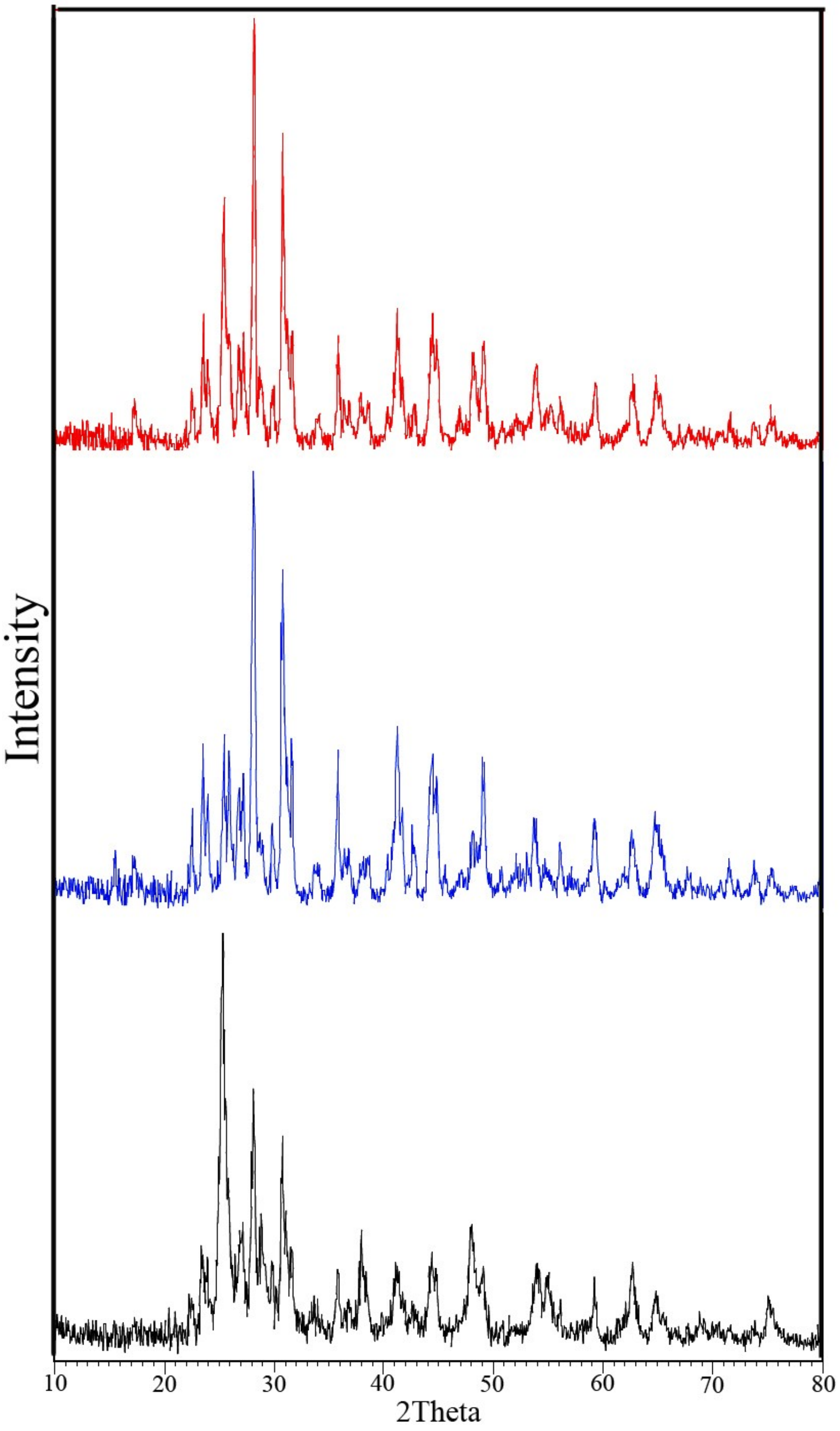

Fig. S3. XRD pattern of sample 1 (a), sample 2 (b), and sample 3 (c) after calcination at 750 °C for 2h.

Table 1. Experimental detail for preparing a typical piezocatalyst.

Sample Number	Reaction Time (h)	Reaction Temperature (℃)	Surfactant
1	6	160	--------
2	6	160	plum
3	6	160	apricot
4	6	160	peach
5	6	160	melon
6	6	140	--------
7	6	180	--------
8	4	160	--------
9	8	160	--------

Table S2. Effect of reaction temperature during synthesis of catalyst on the degradation efficiency of AR151.

Catalyst	Vibration Time (h)	Vibration Power (W)	Pollutant	Degradation Efficiency (%)
T1	2	100	AR151	95.4
T2	2	100	AR151	78.4
T3	2	100	AR151	96.7
